# Optical Sensing Method for Screening Disease in Melon Seeds by Using Optical Coherence Tomography

**DOI:** 10.3390/s111009467

**Published:** 2011-10-10

**Authors:** Changho Lee, Seung-Yeol Lee, Jeong-Yeon Kim, Hee-Young Jung, Jeehyun Kim

**Affiliations:** 1 School of Electrical Engineering and Computer Science, Kyungpook National University, 1370, Sankyuk-dong, Buk-gu, Daegu 702-701, Korea; E-Mail: song31037@knu.ac.kr; 2 School of Applied Biosciences, Kyungpook National University, 1370, Sankyuk-dong, Buk-gu, Daegu 702-701, Korea; E-Mails: leesy1985@gmail.com (S.-Y.L.); heeyoung@knu.ac.kr (H.-Y.J.); 3 Division of General Studies, Ulsan National Institute of Science and Technology, Ulsan 689–798, Korea

**Keywords:** optical sensing, plant imaging

## Abstract

We report a noble optical sensing method to diagnose seed abnormalities using optical coherence tomography (OCT). Melon seeds infected with *Cucumber green mottle mosaic virus* (CGMMV) were scanned by OCT. The cross-sectional sensed area of the abnormal seeds showed an additional subsurface layer under the surface which is not found in normal seeds. The presence of CGMMV in the sample was examined by a blind test (n = 140) and compared by the reverse transcription-polymerase chain reaction. The abnormal layers (n = 40) were quantitatively investigated using A-scan sensing analysis and statistical method. By utilizing 3D OCT image reconstruction, we confirmed the distinctive layers on the whole seeds. These results show that OCT with the proposed data processing method can systemically pick up morphological modification induced by viral infection in seeds, and, furthermore, OCT can play an important role in automatic screening of viral infections in seeds.

## 1. Introduction

*Cucumber green mottle mosaic virus* (CGMMV) was first reported in England in 1953, and an outbreak occurred in Japan in the late 1960s [1]. A CGMMV particle is rod-shaped, 300 nm in length, and 18 nm in width, and is morphologically similar to the *Tobacco mosaic virus* (TMV). CGMMV can currently be identified only by electron microscopy. Disease caused by CGMMV is common in cucumber, melon, muskmelon, and pumpkin. An outbreak of the disease causes considerable economic losses [2]. Viral-diseases are typically diagnosed by a destructive biological or serological sensing method, which require significant effort and time, meaning early noninvasive diagnosis is not feasible. Recently, reverse transcription-polymerase chain reaction (RT-PCR) has been commonly used to sense abnormal plants and seeds that were infected with CGMMV [3,4]. However, this method suffers from several weaknesses, including invasiveness, several hours of processing, and relative high measurement cost per sample. In addition, full number inspection is not a practical choice. Only sampling tests are routinely performed in the field of agriculture.

Several noninvasive diagnostic methods have been imported from medical modalities, such as X-ray tomography [5], positron emission tomography (PET) [6], magnetic resonance imaging (MRI) [7], and ultrasound [8]. These approaches mainly screen the morphological and structural differences in plants, but they also have disadvantages; X-ray tomography and MRI’s image resolution are too low to differentiate several micro-scale structures. PET involves injecting a tracer into the sample, and is not easy to show results in real-time display. Although ultrasound imaging is widely used, it uses an index matching material, such as water or gel, to increase the signal to noise ratio (SNR).

As an alternative optical non-invasive sensor, optical coherence tomography (OCT), developed in 1991 [9], compensates for the limitations of the previous diagnostic techniques. OCT is a noninvasive optical sensing/imaging technique that uses an interferometer to maintain micrometer scaled cross-sectional sensing resolution in samples. OCT shows the structure of the tissue by sensing small changes in back-scattered light at various depths. The scanning depth of OCT is a function of absorption and scattering, and is in order of a few millimeters in biological tissues [10–14]. OCT has become important in several medical fields, including ophthalmology and dermatology because of its real-time imaging capability and micrometer-scale resolution [11,15–17]. The application of OCT has recently expanded to various industrial areas as well, such as security equipment, crack detection, and so on [18,19]. Although OCT images of plants have been reported several times, the purpose was limited to revealing the inner structures in the morphological study of plant tissues in kiwifruit, tomato, spiderwort, and orach [20,21]. Optical coherence microscopy (OCM) has also been applied as a new imaging technique in the plant biology field [22–24]. More recently, in 2010 a pathological morphology in orchid plants and rots in onion were studied using OCT [25,26]. Our study was focused so that OCT with proper data processing can pick up morphological modifications induced by viral infection in seeds, and later OCT can play an important role in the automatic screening of viral infections in seeds.

## 2. Experimental Section

### 2.1. Optical Coherence Tomography

**Figure 1.**
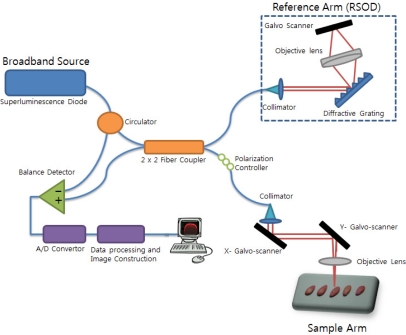
Optical coherence tomography diagram

A schematic diagram of an optical coherence tomography (OCT) system is shown in Figure 1. The OCT light source is a super luminescent emitting diode (SLED) centered at 1310 nm with full width at half maximum (FWHM) of 150 nm. The calculated axial resolution is 4.5 μm in the air. The SLED light enters the circulator, is split into two fibers by a 2 × 2 (50:50) fiber coupler, and the fibers are directed to the reference arm and sample arm of the interferometer. A rapidly scanning optical delay line (RSOD) is used in the reference arm to provide a variable optical pathlength [27]. The RSOD consists of a diffraction grating with 600 grooves/mm and a galvo-scanner that changes the pathlength at 300 Hz. The light in the sample arm is focused by an objective lens with an 18 mm focal length. (Thorlabs LSM02, NA = 0.037). The derived lateral resolution is 13 μm. We used the galvo-scanner in the sample arm to generate the B-scan. The B-scanning range is 2 mm. Backscattered light from the sample is coupled back into the fiber. Interference fringes form at the photodetector when the optical pathlength of the sample and the reference arm are the same. The interference fringe signal is observed only when the pathlengths of the interferometer arms match the coherence length of the source. A balanced photodetector (Model PDB120C-AC; Thorlabs) was adapted to increase the signal to noise-ratio (SNR = 66 dB). The analog signal received from the photodetector is converted to a digital signal by an A/D converter and is stored in text format on a hard drive. The sampling rate is 10,000,000 Hz, the number of samples per single depth scan (A-scan) is 100,000, and the number of A-scans to form a 2-dimensional image is 200. By using X,Y axis galvo-scanners, we acquired 500 number of 2-dimensional images. It took 330 seconds. After finishing 3D scanning, a 3D volume image was obtained by using 3D reconstruction software. The measured axial resolution & lateral resolution of the system in air were 6.7 μm and 17.3 μm, respectively.

### 2.2. Material

**Figure 2.**
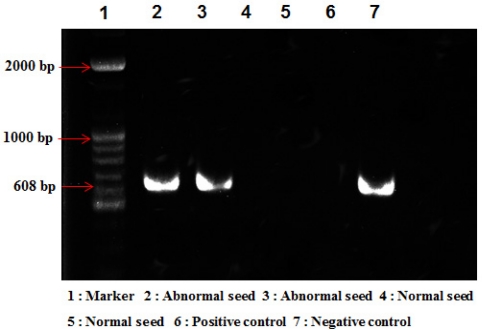
Discrimination between normal and abnormal melon seeds sensed by RT-PCR amplification. PCR products were separated by electrophoresis through a 1% agarose gel. Lane M, 100 base plus ladder DNA; Lane 1 and 2, abnormal seeds; Lane 3 and 4, normal seeds; Lane 5, CGMMV infected melon plant.

Throughout the years 2008 and 2009, 140 units of similarly harvested normal and abnormal melon seeds were collected from Seongju, the largest oriental melon production region in Korea. In spite of the invasiveness of the method, we performed RT-PCR analysis to confirm viral infection in the abnormal seeds. The RT-PCR with universal primers CGMM-C60 (5′-ATT TAA GTA AAG TCC TGA CG-3′) and CGMM-N30 (5′-ATG GAA CGT ACC GGA ATC-3′) was used to amplify the CGMMV movement protein [28]. From several of the abnormal seeds samples examined, the RT-PCR amplified approximately 600 bp fragments, which is the same size as that expected for the CGMMV movement protein (Figure 2 Lane 1–2). However, no amplification products were obtained from the normal seeds under the same conditions (Figure 2 Lane 3–4). From the RT-PCR analysis of seeds, we verified that the provided abnormal melon seeds were indeed inoculated with the CGMMV infection. The sample seeds are used for the OCT image analysis.

## 3. Results and Discussion

### 3.1. Two-Dimensional OCT Images Analysis

**Figure 3.**
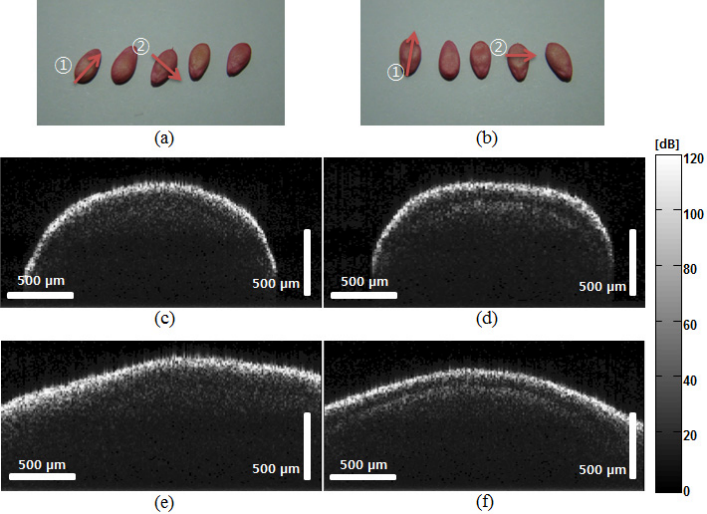
OCT B-mode scan comparison of normal and abnormal melon seeds, (**a**) and (**b**) are pictures of normal and abnormal melon seeds, respectively ( 
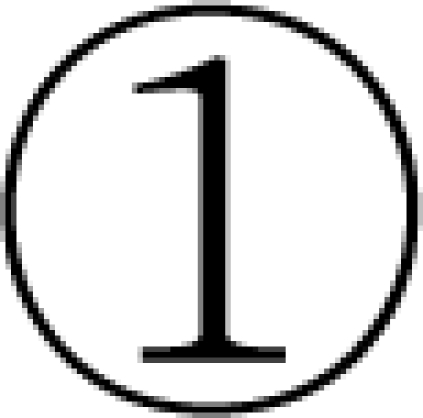
: lateral scan, 
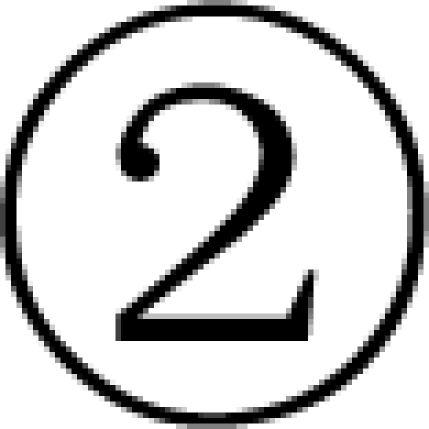
: transverse scan), (**c**) and (**d**) are OCT images of normal and abnormal melon seeds, respectively, in the lateral direction; (**e**) and (**f**) are OCT images of normal and abnormal melon seeds, respectively, in the transverse direction. The scale bars mean in air. The pixel color indicates in a dB scale.

OCT images of normal and abnormal samples are shown in Figure 3. As referenced, five seeds from each sample group were photographed and are shown in [Figure 3(a,b)]. All samples were coated by the collector. The coating was dyed with Food Red 17 that comes as a sodium salt which has fewer health risks. This coating is part of the routine process in handling abnormal seeds and is not considered to alter biological status of the seeds. Morphological change in the OCT image was not observable before/after coating. OCT B-mode scans were generated at a depth of 500 μm and a lateral direction of 2 mm. The B-mode OCT image structure may reflect the unique pattern caused by the scattering property change due to the virus.

Figure 3(c,d) shows images of the samples scanned in the lateral direction, as indicated by arrow 
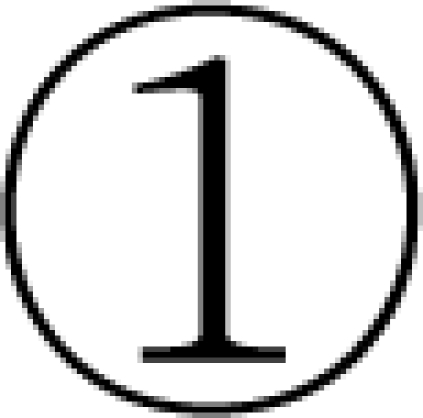
 in Figure 3(a), and Figure 3(e,f) shows images of the samples scanned in the transverse direction. The OCT images of the normal seed [Figure 3(c,e)] contain no distinct layers under the pericarp in either the lateral or the transverse scan. However, a distinct amplitude rise under the pericarp was observed in the OCT images of the abnormal seed [Figure 3(d,f)]. The differentiation in the layer may be caused by the structural modification induced by the viral infection as shown in Figure 6(c). The viral infection may cause an extended (50–100 μm) layer with increased scattering between the aleurone and the endosperm.

A specificity evaluation study was performed by five untrained persons. The total 140 B-scan data, composed of 70 normal and 70 abnormal images, were mixed without any note on the image. The examiners evaluated the test images with their bare eyes. The rates of the true positive for normal and abnormal seeds are 82.85% and 74.85%, respectively.

### 3.2. A-Scan Sensing Analysis & Statistical Analysis

**Figure 4.**
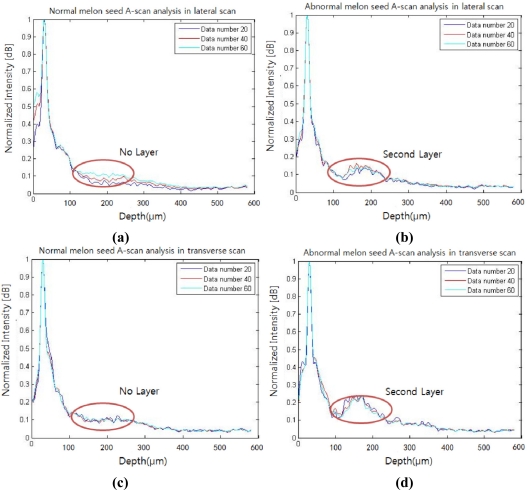
OCT A-scan comparison of normal and abnormal melon seeds. (**a**) and (**b**) are A-scans of normal and abnormal melon seeds, respectively, in the lateral direction; (**c**) and (**d**) are M-mode scans of normal and abnormal melon seeds, respectively, in the transverse direction.

A-scan analysis was performed to provide additional details about both the normal and the abnormal seeds. For the A-scan analysis, the first peaks in the depth direction were searched in an OCT B-mode image, which was later rearranged to flatten the first peaks. In this search, an automated program was coded so that both abrupt intensity variation and intensity peak were decided as the first peak. The 20, 40, and 60 A-scans were sampled and averaged from the middle position of the flattened B-mode image in both lateral and transversal directions. The normalized A-scan analysis is shown in Figure 4. As the number of A-scans average increases, speckles in the results decrease. The profiles of the lateral and transverse scans of the normal seed [Figure 4(a,c)] have no significant peaks other than between air and the pericarp boundary as indicated with red circles. Conversely, the profiles of the lateral and transverse scans of the abnormal seed contain a distinct peak centered near the 100 μm depth from the first peak. The amplitude of the second peak of the lateral scan for the abnormal seed is elevated by 62% from the same position for the normal seed, whereas the transverse scan shows a 120% rise. However, the position of the second peak is consistent in any scanning direction. Based on the measurement of A-scan signal intensity in the Figure 4, each second layer peak intensity in the abnormal and normal seeds were observed as 0.163 ± 0.022 dB, 0.101 ± 0.013 dB, respectively. Both of seed's background signal intensities have a similar level as 0.095 ± 0.02 dB, 0.094 ± 0.014 dB, respectively. When we calculated an intensity difference between the second layer peak and the background signal, the abnormal and normal seeds show the difference as 0.068 ± 0.021 dB, 0.007 ± 0.019 dB, respectively as shown in Table 1.

### 3.3. Three-Dimensional OCT Images Analysis

**Figure 5.**
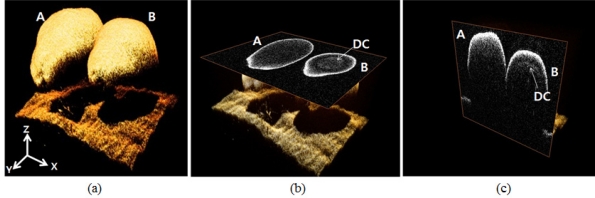
Three dimensional OCT images, (**a**) 3D reconstruction OCT images, (**b**) Enface OCT images, (**c**) Cross-sectional images (A: normal melon seed, B: abnormal melon seed, DC: distinctive layer).

In the two dimensional and A-scan analysis, we only conducted lateral/transverse OCT scans at a specific position of the normal/abnormal seeds, and we arbitrarily selected certain positions to perform further A-scan analysis. To confirm the consistency of the screening over several measurement variations, such as different scanning region, angle, and position in depth, a three dimensional (3D) OCT scan was performed in samples. We reconstructed 3D OCT images by using X and Y galvo-scanners. In Figure 5, 3D images also show that an abnormal melon seed has a distinctive layer covering the whole seed. Figure 5(a) is the 3D rendering OCT image. Figure 5(b) is the enface OCT image. Figure 5(c) is the cross-sectional OCT images. Character A represents the normal melon seed and B is for the abnormal melon seed. DC means the distinctive layer.

### 3.4. Comparison between Micrograph Images and OCT Images

**Figure 6.**
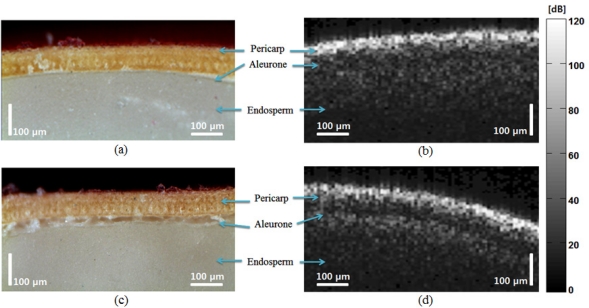
Comparison of OCT image and electron micrographs, (**a**) and (**c**) are an electron micrograph (500×) of normal and abnormal melon seeds, respectively; (**b**) and (**d**) are OCT images of normal and abnormal melon seeds, respectively.

To illustrate the second peak in the abnormal OCT images, we compared the micrograph with the OCT images as shown in Figure 6. The micrograph of the normal seed [Figures 6(a,c)] shows a smooth transition between the aleurone and endosperm; the corresponding OCT signal also shows no distinct peak. The micrograph of the abnormal seed [Figure 6(c)] shows irregular composition in the aleurone layer, which caused distinct peak in the OCT images.

## 4. Conclusions

We have investigated the extended application of OCT to screen abnormal melon seeds infected by CGMMV. Morphological changes in normal and infected seeds were observed by comparing B-mode OCT images. A-scan analysis revealed distinct changes in peaks and slopes in normal and abnormal samples, and 3D OCT images demonstrated that the whole abnormal seed has a distinctive layer under the surface. When we conducted the bare eye experiment on OCT images to distinguish between seventy abnormal and normal melon seeds, the accuracy rate was 82.85% and 74.85% respectively. If the OCT images have an unclear additional layer, it makes it difficult to distinguish the infected seeds precisely.

A weak second boundary was also shown in the normal OCT seed image at the boundary between pericarp and aleurone, but a more distinct change was observed in the abnormal OCT images. The change was quantitatively measured in Table 1. After measuring the A-scan intensity amplitude and then subtracting the background intensity, we can acquire quantitative information about the pure second layer peak amplitude. When we compare the intensity amplitude difference in mixed seeds, the abnormal seed’s value (mean 0.067 dB) is higher than the normal layer’s value (mean 0.007 dB). A threshold value was set to be above 0.03 dB from the OCT signal background intensity (0.095 dB) for an automatic screen. From this result, A-scan analysis data not only shows the possibility that it can be utilized as the standard sheet to screening the infected seeds, but is also a more reliable method than bare eye screening of 2D OCT images.

According to Bennett [29], the presence of the second layer in the abnormal seeds can be explained as one of the most characteristic relationships between plant viruses and their hosts that is the strong protection of embryos against viruses invading the mother plant. This general resistance to seed transmission may result from physical as well as physiological/biochemical barriers to virus entry into reproductive tissues. These barriers which represent specific host-resistance mechanisms may result in the distinctive layer seen in the abnormal seeds. However, the need of further biological study is required to reveal the cause of the irregular composition change in the aleurone. These results suggest the high possibility that OCT will be a promising sensing modality to screen infected melon seeds noninvasively, as well as to monitor any morphological changes in superficial layers caused by abiotic factors or bacterial infections.

## Figures and Tables

**Table 1.** Descriptive statics for OCT A-scan signal intensity measurement of second layer peak signal intensity, background signal intensity, Intensity difference between second layer peak and background signal for the abnormal and the normal seed [n = 20 (abnormal), 20 (normal); NS not significant; Unit: dB].

	
	**Abnormal seeds**		**Normal seeds**		**t-Test**

	**Mean**	**SD**	**Mean**	**SD**	
Second layer peak signal intensity	0.163	0.022	0.101	0.013	NS (P = 0.001)
Background signal intensity	0.095	0.020	0.094	0.014	0.885 (P ≤ 0.44)
Intensity difference	0.068	0.021	0.007	0.019	NS (P = 0.001)
